# Gut microbiota composition is associated with polypharmacy in elderly hospitalized patients

**DOI:** 10.1038/s41598-017-10734-y

**Published:** 2017-09-11

**Authors:** Andrea Ticinesi, Christian Milani, Fulvio Lauretani, Antonio Nouvenne, Leonardo Mancabelli, Gabriele Andrea Lugli, Francesca Turroni, Sabrina Duranti, Marta Mangifesta, Alice Viappiani, Chiara Ferrario, Marcello Maggio, Marco Ventura, Tiziana Meschi

**Affiliations:** 1 Department of Medicine and Surgery, University of Parma, Parma, Italy; 2 Dipartimento Medico-Geriatrico-Riabilitativo, Azienda Ospedaliero-Universitaria di Parma, Parma, Italy; 3 Laboratory of Probiogenomics, Department of Chemistry, Life Sciences and Environmental Sustainability, University of Parma, Parma, Italy; 4 GenProbio S.r.l., Parma, Italy

## Abstract

Reduced biodiversity and increased representation of opportunistic pathogens are typical features of gut microbiota composition in aging. Few studies have investigated their correlation with polypharmacy, multimorbidity and frailty. To assess it, we analyzed the fecal microbiota from 76 inpatients, aged 83 ± 8. Microbiome biodiversity (Chao1 index) and relative abundance of individual bacterial taxa were determined by next-generation 16S rRNA microbial profiling. Their correlation with number of drugs, and indexes of multimorbidity and frailty were verified using multivariate linear regression models. The impact of gut microbiota biodiversity on mortality, rehospitalizations and incident sepsis was also assessed after a 2-year follow-up, using Cox regression analysis. We found a significant negative correlation between the number of drugs and Chao1 Index at multivariate analysis. The number of drugs was associated with the average relative abundance of 15 taxa. The drug classes exhibiting the strongest association with single taxa abundance were proton pump inhibitors, antidepressants and antipsychotics. Conversely, frailty and multimorbidity were not significantly associated with gut microbiota biodiversity. Very low Chao1 index was also a significant predictor of mortality, but not of rehospitalizations and sepsis, at follow-up. In aging, polypharmacy may thus represent a determinant of gut microbiota composition, with detrimental clinical consequences.

## Introduction

Human gut microbiota composition, which is relatively stable during the adult lifespan, faces significant alterations with aging^1, 2^. Most studies have emphasized the age-related reduction in biodiversity^3–5^, which is more pronounced in centenarians^6, 7^ and in subjects living in nursing homes^3, 8^.

For example, in a cohort of 178 Irish older individuals, institutionalization was associated with a reduced representation of short-chain fatty acids (SCFA) producers and expansion of taxa including *Parabacteroides*, *Eubacterium*, *Coprococcus* and *Anaerotruncus* in gut microbiota^3^. These alterations may partly depend on different dietary patterns followed by elderly living in nursing homes, given the strong influence of diet on gut microbiota composition^9^. However, recent studies have demonstrated that most of these alterations are independent of nutrient intake^8^. These specific patterns, in the absence of acute diseases, remain relatively stable over time^10^.

Gut microbiota composition in the elderly may have important physio-pathological implications, since in aged murine models the gut dysbiosis promotes chronic inflammation and is associated with a reduced innate immunity function^11^. Moreover, depletion of taxa producing SCFA may promote anabolic resistance^12^.

To date, very few studies have investigated the possible association of human gut microbiota composition with clinical and functional parameters in aging. In a subgroup of participants to the Eldermet Study, Jeffery and colleagues identified a group of microbial taxa, including *Anaerotruncus*, *Desulfovibrio* and *Coprobacillus*, whose relative abundance in the gut was inversely correlated with Barthel Index^8^. Similarly, in a cohort of 364 couples of elderly female twins, Jackson *et al*. identified 21 gut microbial taxa whose relative abundance was significantly associated with the Rockwood Frailty Index^13^. These findings allow to hypothesize that frailty is associated to a specific microbiota composition.

However, the association of gut microbiota composition with multimorbidity and polypharmacy has not been investigated yet, even if the current literature state of art supports the hypothesis that gut dysbiosis influences the pattern of aging and pathophysiology of some diseases^14^. This is especially true in the hospital setting, where dysbiosis is frequently detected in older patients^15^.

In the present study, we analyzed the fecal microbiota composition of 76 elderly patients without gastrointestinal diseases hospitalized in a large Italian geriatric ward. The aim was to assess the possible association of gut microbiota composition, determined through metagenomics analysis (16S rRNA microbial profiling), with the domains of frailty, multimorbidity and polypharmacy, and to elucidate the consequences of a reduced biodiversity on clinical outcomes.

## Methods

### Study design and population

With an observational study design, we enrolled 76 elderly (age ≥ 65) multimorbid (≥2 chronic diseases) patients who were acutely hospitalized to the Internal Medicine ward of Parma University Hospital Geriatric-Rehabilitation Department, in Northern Italy, from November 2014 to April 2015. Enrolled patients were all urgently admitted from the Emergency Department and suffered from acute extra-intestinal diseases and a high burden of multimorbidity, in line with the case-mix of the ward^16^.

Excluded from the study were subjects with acute or chronic digestive diseases, including inflammatory bowel disease, intestinal ischemia, diverticulitis, cirrhosis, acute or chronic pancreatitis, cholecystitis, intestinal cancer, or with previous abdominal surgery, and subjects who were treated with antibiotics for more than 3 days. Similarly, subjects with expected survival < 30 days or undergoing artificial nutrition were also excluded. In fact, all these conditions have an established association with gut dysbiosis^17^. Conversely, subjects exposed to antibiotic treatment for less than 3 days at the time of enrolment were included in the study, since in older hospitalized patients^15^ and in other settings^10^ short-term antibiotic therapy was not associated with significant alterations in overall microbiota composition.

Moreover, another group of 25 healthy active elderly volunteers (age ≥ 65), without multimorbidity (no more than one chronic illness) and frailty and not taking polypharmacy (<5 drugs), was enrolled in the study on an outpatient basis as control group. None of the volunteers had diseases known to influence fecal microbiota composition or underwent systemic antibiotic treatment in the month before the evaluation.

The protocol followed the principles of the Declaration of Helsinki, and all participants gave their informed consent. The Ethics Committee of the University of Parma approved the study (ID 14091).

### Data collection and laboratory analyses

Hospitalized patients were enrolled within 72 hours from hospital admission. Data about main diagnosis, comorbidities, number and type of medications, functional performance and weight were collected for each patient. The multimorbidity burden was assessed through the Cumulative Illness Rating Scale (CIRS) model, and the CIRS Comorbidity Score was calculated^18^. This score, ranging from 0 to 56, is the sum of the individual indexes of disease severity, ranging from 0 (absence of disease) to 4 (life-threatening condition), assigned to 14 items representing possible organs affected by an acute or chronic disease. The functional performance of participants was assessed according to the Rockwood Clinical Frailty Scale (RCFS)^19^. Polypharmacy was assessed through a systematic recognition of drugs chronically taken by every patient.

Within the first 72 hours of hospital stay, each patient provided a stool sample of at least 2 grams by spontaneous evacuation. All patients followed the same hospital-based oral diet at the time of stool sample collection, characterized by balanced intake of energy (1700 Kcal/day) and macronutrients. Fecal samples were immediately frozen at −20 °C and delivered to the Laboratory of Probiogenomics of Parma University for processing and 16S rRNA microbial profiling analyses.

Control subjects underwent a general clinical examination, to assess eligibility for inclusion in the study. Each of them provided a stool sample, which was handled in the same way as samples of hospitalized subjects.

The laboratory procedures and bioinformatics analyses were performed according to a validated protocol^15, 20^, which is fully described elsewhere^20^ and summarized in the Supplemental Material. The bacterial 16S rRNA reads were analyzed with the QIIME software^21^ and pooled in Operational Taxonomic Units (OTUs) with the uclust software^22^. The OTU data were used to calculate the index of biodiversity Chao1 and to perform a beta diversity analysis with the Principal Coordinate Analysis (PCoA) method based on unweighted UniFrax. Taxonomic classification of OTUs was made using the QIIME software suite^21^ and the related 16S database SILVA^23^, and the relative abundance of every taxon was determined in all samples.

### Evaluation of clinical outcomes

In order to prospectively verify the clinical effect of a reduced fecal microbiota biodiversity, hospitalized patients were actively followed-up, as part of the normal clinical procedures adopted in our institution. Thus, data about mortality and rehospitalizations were collected for two years after discharge. Namely, the dates of death or hospital readmission were considered. In case of hospital readmission, the presence of sepsis was also investigated as outcome. In fact, epidemiological studies have demonstrated an association between gut dysbiosis and risk of sepsis^24^.

### Statistical analyses

Clinical data and alpha-diversity indexes were expressed as average ± standard deviation or, for non-normally skewed distributions, median and tertiles or quartiles. The relative abundance of single taxa within the gut microbiota of every patient was expressed as percentage on the whole number of bacteria detected by metagenomics analyses.

The overall fecal microbiota composition, in terms of inter-individual variability, was compared between hospitalized patients and healthy controls using Permanova and Kendall tau-rank co-occurrence analysis.

In hospitalized patients, Pearson correlation analysis was carried out to test the association between each of the clinical (CIRS Comorbidity Score, RCFS, number of drugs) and microbial biodiversity variables (Chao1 index, relative abundance of single taxa). In multivariate linear regression models, age, sex, exposure to antibiotics and number of days under antibiotic treatment were considered as potential confounders. The average Chao1 index was also compared between the top and the bottom tertile of CIRS Comorbidity Score, RCFS and number of drugs using a one-way Kruskal-Wallis analysis of variance adjusted for age, sex and antibiotic exposure. Finally, an explorative analysis with multivariate linear regression was carried out to test the possible association between the relative abundance of taxa significantly related to polypharmacy and exposure to specific drug classes, i.e. diuretics, proton-pump inhibitors (PPIs), neuroleptics, antidepressants, acetaminophen, antinflammatory drugs, antihypertensive and lipid-lowering treatments.

Finally, in hospitalized patients, univariate and multivariate models of Cox regression analysis were built to assess the association between fecal microbiota biodiversity, measured with the Chao1 index, and clinical outcomes, i.e. two-year mortality, rehospitalization, and incident sepsis. Kaplan-Meier survival analysis was also performed after categorization of patients in tertiles of Chao1 index.

Statistical analyses were carried out using QSPSS and the SAS statistical package, version 9.1 (SAS Institute Inc., Cary, North Carolina), considering p values ≤ 0.05 as significant.

### Data availability statement

The 16S rRNA profiling data sequenced in this study, together with essential clinical data, were deposited in SRA database under the following accession numbers: PRJNA297268 and PRJNA297269.

## Results

### Clinical features of hospitalized patients

The 76 hospitalized patients (39 M, 37 F) enrolled in this study were 83 ± 8 years old and displayed a high burden of chronic multimorbidity (CIRS Comorbidity Score median 11, first tertile ≤ 7, second tertile 8–14, third tertile ≥ 15), frailty (RCFS median 5, first tertile ≤ 4, second tertile 5–6, third tertile ≥ 7) and polypharmacy (number of drugs median 10, first tertile ≤ 7, second tertile 8–10, third tertile ≥ 11 drugs). Forty patients out of 76 (52.6%) were taking antibiotic treatment at the time of stool sample collection, but in no cases this treatment had been lasting for more than 72 hours. The most prevalent chronic comorbidities are listed in Table 1. The most frequent reasons for hospital admission were pneumonia, COPD with respiratory failure, congestive heart failure, arrhythmic syncope, fall-related trauma, ischemic stroke.

**Table 1:** Clinical metadata. Main characteristics of hospitalized patients enrolled in the study (n = 76).

Age (years, mean ± SD)	83.3 ± 7.5
Female sex (n, %)	37 (48)
Weight (kg, mean ± SD)	70.3 ± 18.4
Number of drugs (n, median and IQR)	10 [8–12]
CIRS Comorbidity Score (points, median and IQR)	11 [8–14.5]
Rockwood Clinical Frailty Scale (rank, median and IQR)	5 [4–7]
Length of antibiotic exposure (days, median and IQR)	1 [0–2]
Length of hospital stay (days, median and IQR)	7 [4.5–20]
Number of chronic comorbidities (n, median and IQR)	5 [3–6]
Prevalence of dementia (n, %)	27 (35)
Prevalence of neurological diseases other than dementia (n, %)	17 (22)
Prevalence of hypertension (n, %)	49 (63)
Prevalence of cardiovascular diseases (n, %)	40 (52)
Prevalence of respiratory diseases (n, %)	19 (25)
Prevalence of diabetes, (n, %)	24 (31)
Prevalence of chronic renal failure, (n, %)	14 (19)
White Blood Cells, (u/mm^3^, median and IQR)	8270 [6460–11130]
C-reactive protein (mg/L, median and IQR)	80 [21–140]
Hemoglobin (g/dl, median and IQR)	11.9 [9.8–13.3]
Creatinine (mg/dl, median and IQR)	1.0 [0.8–1.4]
Sodium (mEq/L, median and IQR)	139 [137–141]
Potassium (mEq/L, median and IQR)	4.1 [3.7–4.4]
Albumin (g/dl, median and IQR)	3.3 [3.0–3.6]
Total cholesterol (mg/dl, median and IQR)	151 [126–179]
Triglycerides (mg/dl, median and IQR)	93 [75–124]

According to the Rockwood model, the prevalence of frailty-disability (RCFS 5–9), pre-frailty (RCFS 4) and fitness (RCFS 1–3) were 57%, 22% and 21%, respectively. The prevalence of polypharmacy (≥5 drugs chronically taken before hospitalization) was 94%. The most frequent drug classes were PPIs (73%), antihypertensives (63%) and diuretics (60%).

The main clinical features of hospitalized participants are summarized in Table 1.

### Comparison of fecal microbiota composition between hospitalized patients and controls

**Figure 1:**
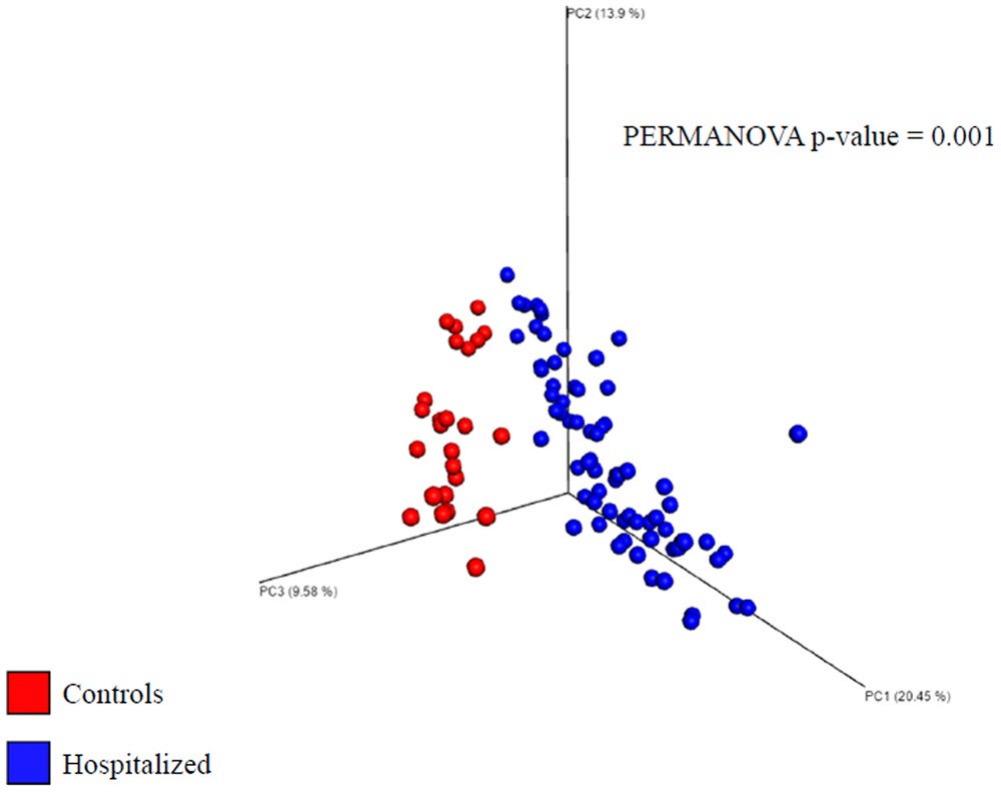
Gut microbiota in cases and controls. Comparison of the overall fecal microbiota composition, represented with 3D Principal Coordinate Analysis scatterplot, between 76 older hospitalized patients with a high burden of multimorbidity, frailty and polypharmacy, and 25 older healthy-active volunteers without polypharmacy.

Hospitalized patients exhibited a significantly different fecal microbiota composition, in terms of beta-diversity, than controls enrolled on an outpatient basis (Permanova p = 0.001). The Principal Coordinate Analysis (PCoA) based on unweighed UniFrac showed two distinct microbiome clusters, corresponding to hospitalized patients and older controls (Fig. 1).

### Gut microbiota biodiversity and clinical domains in hospitalized subjects

**Figure 2:**
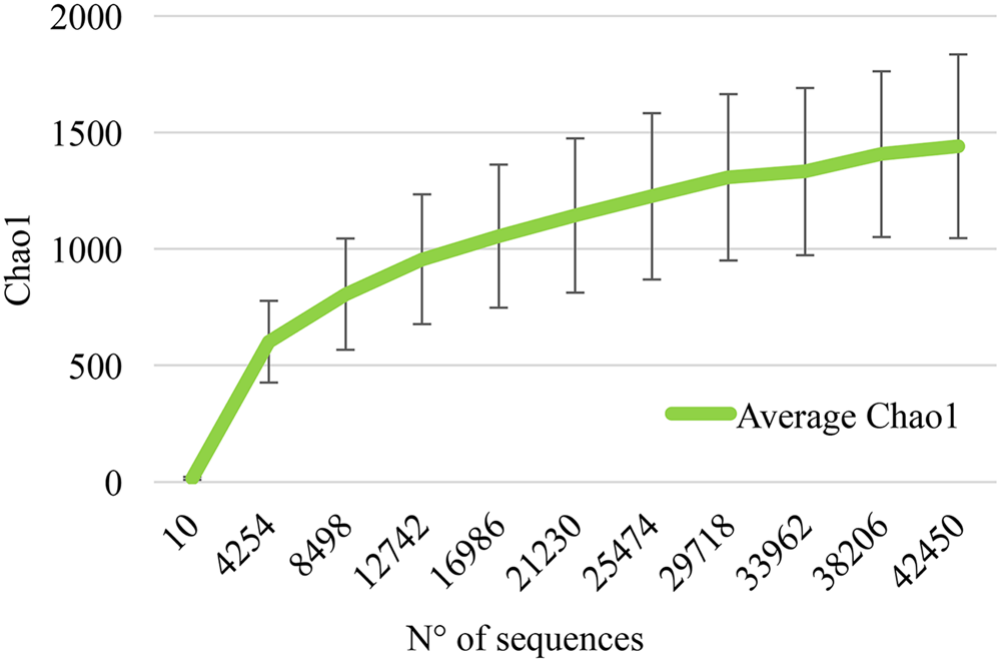
Average gut microbiota biodiversity of hospitalized patients. Average gut microbiota alpha-diversity curve of the 76 stool samples analyzed by 16S rRNA microbial profiling metagenomics techniques. The curve represents the average Chao1 index, corresponding to the number of Operational Taxonomic Units (OTUs), at increasing sequencing depth.

The 16S rRNA microbial profiling analysis of the 76 stool samples, collected while patients followed the same hospital-based diet, produced a total of 3470225 filtered reads, with an average of 45661 per sample, corresponding to an average number of 888 OTUs. The average alpha-diversity curve, determined through the Chao1 index, is depicted in Fig. 2.

At a univariate Pearson analysis, the Chao1 index was significantly and negatively correlated with the number of drugs (r = −0.278, p = 0.018), but not with the CIRS Comorbidity Score (r = 0.015, p = 0.899) and RCFS (r = −0.073, p = 0.570). At a multivariate linear regression model, using backward selection, accounting for age, sex, CIRS Comorbidity Score, RCFS and days of exposure to antibiotic treatment, the negative correlation between number of drugs and Chao1 index persisted as statistically significant (β ± SE −28.54 ± 9.88, p = 0.005) (Table 2).

**Table 2:** Association of gut microbiota biodiversity with clinical domains in hospitalized patients. Multivariate linear regression model, obtained by backward selection analysis of covariates, testing the possible correlation of Chao1 index of alpha-diversity (biodiversity) in gut microbiota with clinical domains in 76 elderly patients hospitalized for acute extra-intestinal diseases.

**Clinical variables**	**Beta ± SE**	**p**
Number of drugs	−28.54 ± 9.88	**0.005**
Rockwood Clinical Frailty Scale	−14.18 ± 2.18	0.51
CIRS Comorbidity Score	4.91 ± 9.06	0.58
Age	9.17 ± 4.83	0.06
Sex (female vs male)	2.14 ± 7.25	0.97
Days of antibiotic exposure	−128.76 ± 84.27	0.13

SE = Standard Error; CIRS = Cumulative Illness Rating Scale

Significant p values (≤0.05) are indicated in bold.

**Figure 3:**
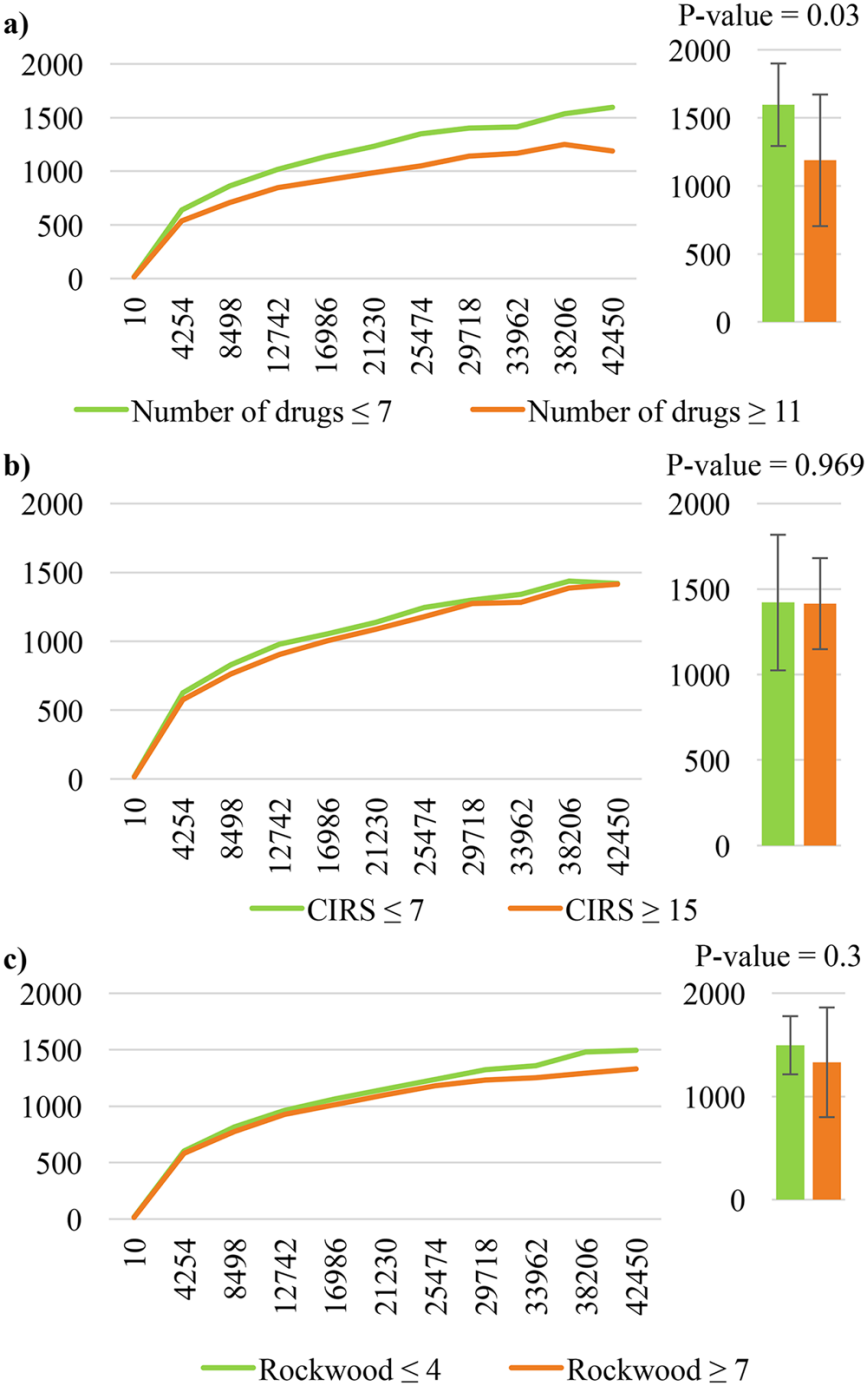
Gut microbiota biodiversity and polypharmacy, multimorbidity and frailty. Comparison of the average gut microbiota alpha-diversity curves, representing Chao1 indexes of biodiversity as function of sequencing depth, between the top and the bottom tertile of 76 patients categorized for (**a**) number of drugs, (**b**) Cumulative Illness Rating Scale (CIRS) Comorbidity Score, and (**c**) Rockwood Clinical Frailty Scale.

As shown in Fig. 3a, comparison of the alpha diversity Chao1 curves between those patients in the top tertile of number of drugs (i.e., chronically taking ≥ 11 drugs) and patients in the bottom tertile (i.e., chronically taking ≤7 drugs) revealed a statistically significant difference (p for trend = 0.03 at different sequencing depths). This difference persisted even after correction for age, sex, CIRS Comorbidity Score and RCFS (p for trend = 0.04 at different sequencing depths). Conversely, similar analyses carried out comparing patients in the top vs the bottom tertile of CIRS Comorbidity Score (≥15 vs ≤7) (Fig. 3b) and patients in the top vs the bottom tertile of RCFS (≥7 vs ≤ 4) (Fig. 3c) did not show any statistical difference (p = 0.969 and p = 0.300, respectively).

### Gut microbiota inter-individual variability and clinical domains in hospitalized patients

**Figure 4:**
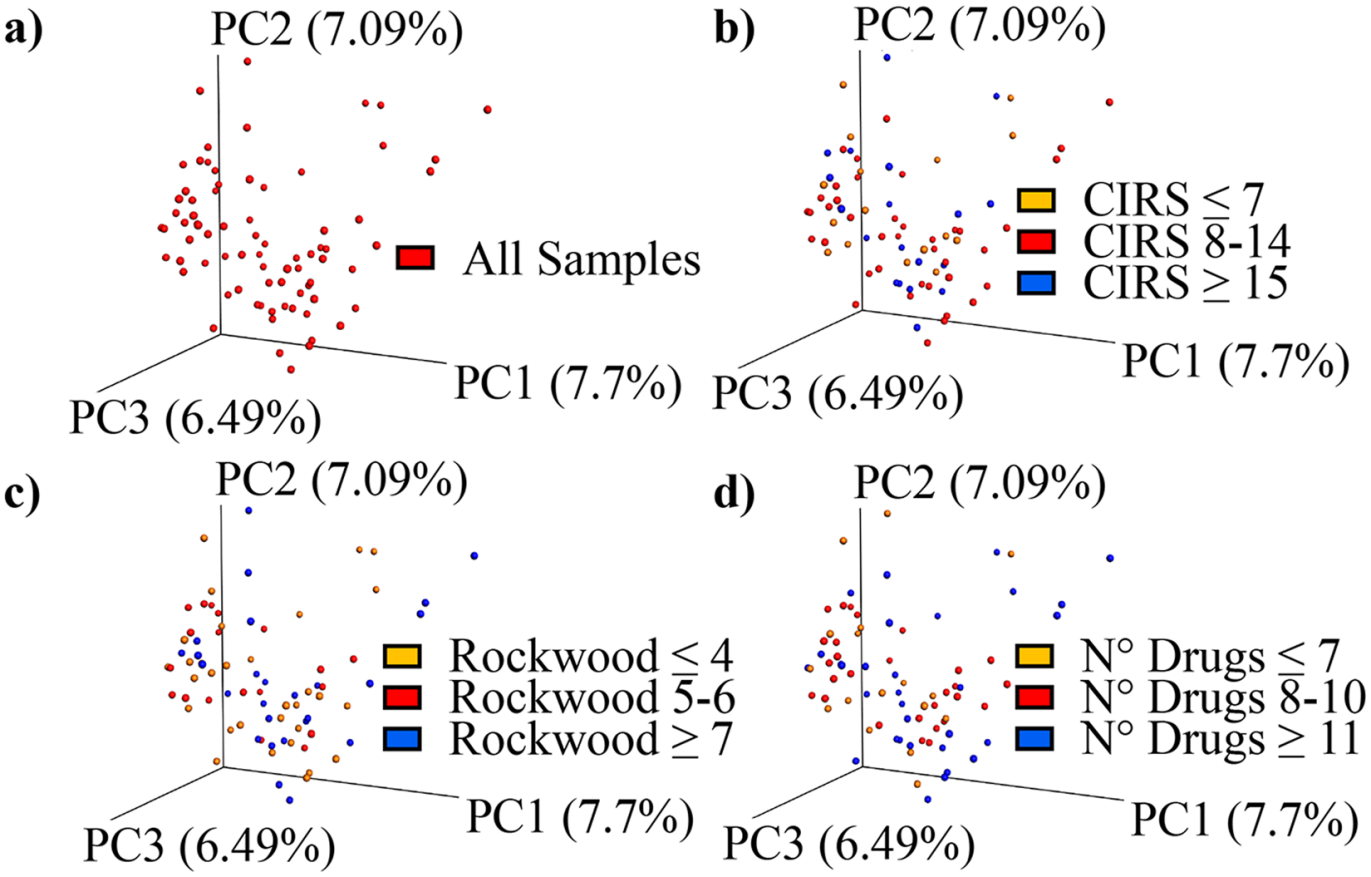
Inter-individual variability of gut microbiota in hospitalized patients and clinical domains. Beta-diversity analysis of inter-individual variability within the 76 stool samples of hospitalized patients, performed with the PCoA method based on an unweighted UniFrax matrix. (**a**) All samples; (**b**) samples categorized for Cumulative Illness Rating Scale (CIRS) comorbidity score tertiles; (**c**) samples categorized for Rockwood Clinical Frailty Scale tertiles; (**d**) samples categorized for number of drugs tertiles.

Beta-diversity analysis of inter-individual variability, performed with the PCoA method based on an unweighted UniFrax matrix, showed no identifiable microbiota clusters, even after categorization of stool samples for tertiles of number of drugs, CIRS Comorbidity Score, and RCFS (Fig. 4). PCA scatter plots obtained categorizing patients according to chronic exposure to specific drug treatments are shown in Supplemental Material. No significant clusters could be identified as well.

### Association of single taxa with clinical domains in hospitalized patients

The overall composition of gut microbiota at genus level for each sample analyzed is depicted in Supplemental Fig. 1. The possible association of the relative abundance of each detected microbial taxon with the considered clinical variables (number of drugs, RCFS, CIRS Comorbidity Score) was tested in Pearson correlation models, whose results are shown in Supplemental Material. For each taxon, multivariate models were then built, considering also the length of antibiotic exposure as potential confounder.

In these multivariate models, the relative abundance of fifteen taxa was significantly associated (p ≤ 0.05) with the number of drugs (Table 3). The association was negative for four taxa, including *Massilia* and unclassified member of *Lachnospiraceae* family, and positive for eleven taxa, including *Bradyrhizobium*, *Coprobacter*, *Helicobacter* and *Prevotella*.

**Table 3:** Association of the relative abundance of single gut bacteria with clinical domains in hospitalized patients. Significant correlations between relative abundance of specific taxa of gut microbiota, identified by 16S rRNA microbial profiling analyses, and clinical parameters (number of drugs, Rockwood Clinical Frailty Scale and CIRS Comorbidity Score) at multivariate linear regression models. Taxa identified in the gut microbiota but lacking any significant correlation with the considered clinical parameters are not shown, but are fully listed in Supplemental Material.

***Taxa***	**Number of drugs**		**Rockwood Clinical Frailty Scale**		**CIRS Comorbidity Score**	
	**Beta ± SE**	p*	**Beta ± SE**	p**	**Beta ± SE**	p***
*Asteroleplasma*	0.0008 ± 0.00004	0.03	—	—	—	—
*Bradyrhizobium*	0.000002 ± 0.0000001	0.02	—	—	—	—
*Coprobacter*	0.0005 ± 0.0002	0.04	—	—	—	—
*Enterobacter*	—	—	—	—	0.00003 ± 0.00001	0.02
*Fonticella*	—	—	0.0009 ± 0.0004	0.04	—	—
*Gordonibacter*	0.00003 ± 0.00001	0.04	—	—	—	—
*Helicobacter*	0.0005 ± 0.00002	0.003	—	—	—	—
*Massilia*	−0.000007 ± 0.000003	0.04	—	—	—	—
*Morganella*	—	—	—	—	0.00004 ± 0.00001	0.004
*Oscillospira*	—	—	0.0005 ± 0.00002	0.02	—	—
*Parabacteroides*	0.003 ± 0.001	0.02	—	—	—	—
*Peptococcus*	—	—	0.00001 ± 0.000006	0.02	—	—
*Porphyromonas*	—	—	0.0001 ± 0.00005	0.02	—	—
*Prevotella*	0.003 ± 0.0001	0.01	0.00001 ± 0.000006	0.02	—	—
*Pantoea*	—	—	—	—	0.0002 ± 0.00008	0.008
*Roseburia*	—	—	—	—	−0.00003 ± 0.00001	0.02
*Scardovia*	—	—	—	—	0.00002 ± 0.000008	0.003
*Shuttleworthia*	—	—	—	—	−0.002 ± 0.001	0.04
U. m. of candidate division TM7 *phylum*	—	—	—	—	0.000001 ± 0.000001	0.01
U. m. of *Bacilli* class	−0.00004 ± 0.000001	0.02	—	—	—	—
U. m. of *Clostridiaceae* 1 family	—	—	0.00003 ± 0.00001	0.04	—	—
U. m. of *Lachnospiraceae* family	−0.13 ± 0.006	0.03	0.01 ± 0.006	0.04	—	—
U. m. of *Ruminococcaceae* family	0.005 ± 0.002	0.02	—	—	—	—
U. m. of *Clostridia* class	—	—	—	—	0.000003 ± 0.000002	0.05
U. m. of *Erysipelotrichiaceae* family 1	—	—	—	—	−0.0007 ± 0.00004	0.05
U. m. of *Erysipelotrichiaceae* family 2	—	—	—	—	0.00004 ± 0.00001	<0.001
U. m. of A0839 family	0.0001 ± 0.00004	0.01	—	—	—	—
U. m. of *Desulfovibrionaceae* family	0.000001 ± 0.0000001	0.01	—	—	—	—
U. m. of *Succinivibrionaceae* family	−0.000006 ± 0.0000003	0.05	—	—	—	—
*Victivallis*	0.00006 ± 0.00003	0.04	—	—	—	—

U. m. = Unclassified member; SE = Standard Error.

p* = Multivariate linear regression models adjusted for age, sex, Rockwood Clinical Frailty Scale, CIRS Comorbidity Score and length of antibiotic exposure.

p** = Multivariate linear regression models adjusted for age, sex, number of drugs, CIRS Comorbidity Score and length of antibiotic exposure.

p*** = Multivariate linear regression models adjusted for age, sex, number of drugs, Rockwood Clinical Frailty Scale and length of antibiotic exposure.

Moreover, the relative abundance of seven taxa was significantly and positively associated (p ≤ 0.05) with RCFS (Table 2), and the relative abundance of ten taxa was significantly associated (p ≤ 0.05) with CIRS Comorbidity Score (Table 2).

### Association of single taxa with specific drugs in hospitalized patients

For the fifteen taxa whose relative abundance in gut microbiota was significantly associated with the number of drugs, we tested whether the relative abundance was associated to exposure to specific drug classes. The significant results of multivariate models, considering age, sex, RCFS, number of drugs, CIRS Comorbidity Score, days of antibiotic treatment and exposure to specific drug classes, i.e., diuretics, proton-pump inhibitors (PPIs), neuroleptics, antidepressants, acetaminophen, antinflammatory drugs, antihypertensive and lipid-lowering drugs, as potential confounders, are shown in Table 4.

**Table 4:** Association of the relative abundance of single gut bacteria with specific drug classes in hospitalized patients. Significant correlations between relative abundance of specific taxa of gut microbiota, identified by 16S rRNA microbial profiling analyses, and exposure to specific drugs classes at multivariate linear regression models. Taxa whose relative abundance was not significantly correlated with any specific drug treatment considered in the analysis (diuretics, proton-pump inhibitors, neuroleptics, antidepressants, acetaminophen, antinflammatory drugs, antihypertensive and lipid-lowering drugs) are not shown.

***Taxa***	**Antidepressants**		**Antipsychotics**		**PPIs**	
	**Beta ± SE**	p*	**Beta ± SE**	p*	**Beta ± SE**	p*
*Asteroleplasma*	0.0007 ± 0.00004	0.04	—	—	—	—
*Helicobacter*	0.003 ± 0.001	0.04	—	—	—	—
*Marinilactibacillus*	0.0002 ± 0.00008	0.03	—	—	—	—
*Prevotella*	—	—	0.04 ± 0.01	0.02	—	—
U. m. of *Bacilli* class	0.0001 ± 0.000009	0.04	—	—	—	—
U. m. of *Desulfovibrionaceae* family	—	—	0.000007 ± 0.000002	0.005	0.00001 ± 0.0000001	0.01
U. m. of *Succinivibrionaceae* family	0.00004 ± 0.00009	0.04	—	—	—	—
*Victivallis*	—	—	0.0002 ± 0.0001	0.04	0.0002 ± 0.0001	0.04

U. m. = Unclassified member; SE = Standard Error; PPIs = Proton Pump Inhibitors.

p* = Multivariate linear regression models adjusted for age, sex, number of drugs, Rockwood Clinical Frailty Scale, CIRS Comorbidity Score, length of antibiotic exposure and other drug classes.

### Fecal microbiota biodiversity and clinical outcomes in hospitalized patients

After a two-year follow-up period, 48 patients (63%) had died, 43 had been rehospitalized (56%), and 18 had had at least one incident episode of sepsis (24%). At univariate and multivariate models of Cox regression analysis, Chao1 index of fecal microbiota biodiversity < 1105, corresponding to the 66th percentile, was a significant predictor of death (RR 2.56, 95% CI 1.21–5.96, p = 0.012 adjusted for age, sex, CIRS Comorbidity Score, RCFS and number of drugs), but not of rehospitalization (RR 1.00, 95% CI 0.99–1.03, age- and sex-adjusted p = 0.43) or sepsis (RR 0.99, 95% CI 0.99–1.02, age- and sex-adjusted p = 0.29).

**Figure 5:**
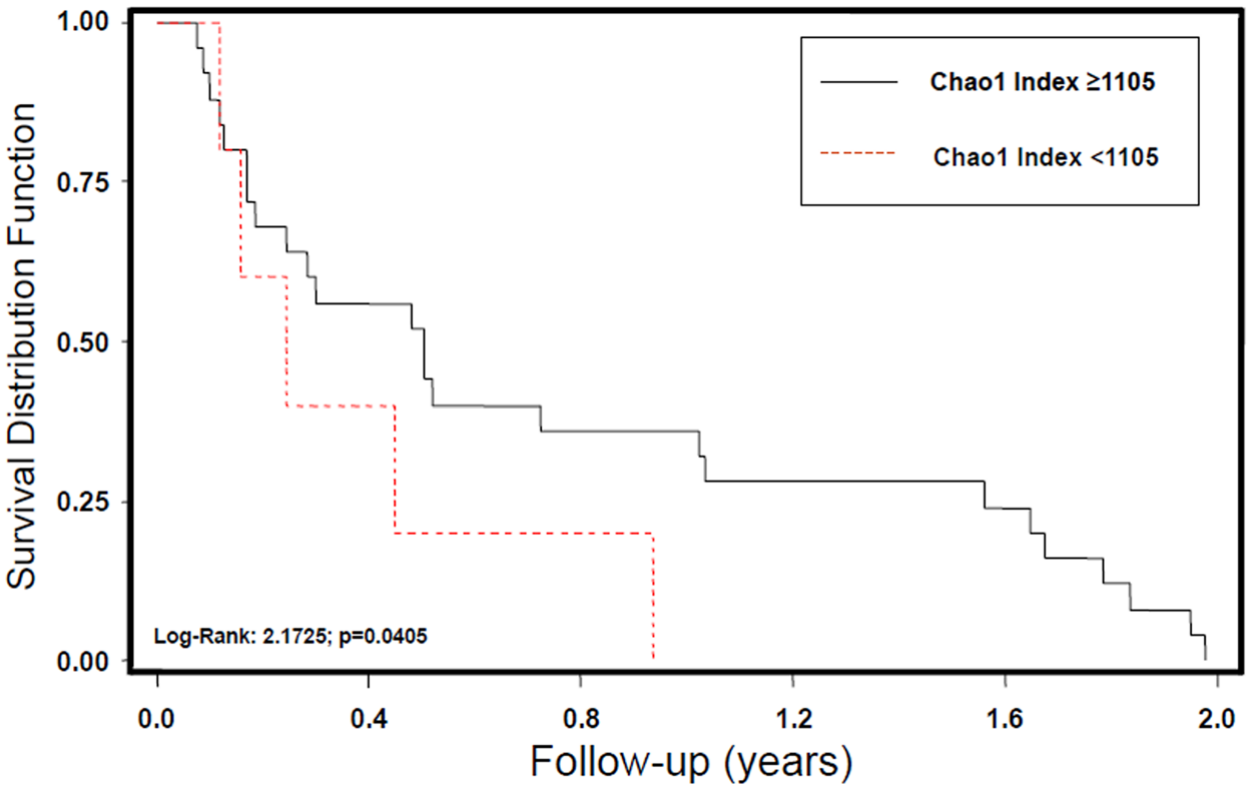
Fecal microbiota biodiversity and survival in hospitalized patients. Survival distribution function of 76 hospitalized patients categorized according to values of Chao1 Index of biodiversity in fecal microbiota. Subjects with higher biodiversity (upper tertile of Chao1 Index, values ≥ 1105) have a statistically longer survival than patients with deeper dysbiosis after a 2-year follow-up.

Kaplan-Meier analysis, depicted in Fig. 5, confirmed that fecal microbiota dysbiosis, with Chao1 index < 1105, was associated with a significantly reduced survival (log-rank 2.1725, p = 0.045).

## Discussion

In this study, we have demonstrated that, in a group of older hospitalized patients, polypharmacy was significantly associated with gut microbiota dysbiosis, i.e. reduction in species richness and significant variations in the average relative abundance of a large number of taxa, including *Helicobacter*. Dysbiosis also exhibited a significant association with mortality at follow-up. Healthy-active older subjects without polypharmacy did not instead exhibit dysbiosis.

Among specific drug classes, PPIs, antipsychotics and antidepressants had the strongest associations with gut microbiota composition. Conversely, indexes of multimorbidity and frailty/disability were not significantly associated with dysbiosis, albeit the average relative abundance of some taxa, including the well-known *Prevotella* and *Enterobacter*, was significantly associated with these indexes.

These results represent the first demonstration to date of the association between polypharmacy and gut microbiota composition, at both quantitative and qualitative level, in a group of elderly hospitalized patients following the same dietary regimen. Thus, this could be an important basis for designing future research aimed at exploring the possible interconnections between gut microbiota composition and (un)successful aging, and for better understanding the pathophysiology of multiple drug treatments.

However, some study limitations should be considered. First, the studied population had a high burden of chronic diseases and disability, whose impact on gut microbiota composition may not be completely measured and explained by the considered indexes. For the same reason, the sample size could have been insufficient to fully represent the high inter-individual variability of microbiota, and could have influenced the results. Second, the studied population had different admission diagnoses. The association of some of these acute diseases, such as pneumonia, congestive heart failure, and stroke, with gut microbiota has been hypothesized^25^, but not verified in population-based studies^26^. Even if subjects with diseases known to have an established association with dysbiosis, such as IBD, were excluded from the study, and no significant microbiome clusters were identified among subjects with the same acute disease, the population heterogeneity may have represented a possible source of bias. Third, other factors explaining gut microbiota variability in adults, such as long-term dietary habits and smoking^26^, were not considered in the present study. For all these reasons, our results should be considered as hypothesis generator for future research rather than definitive remarks on the topic. Future research should also verify whether fecal microbiota composition changes with medication revision in older patients.

The concept that gut microbiota composition may be influenced by drug therapy and drug-drug interactions has already been proposed by some authors^26–28^. However, all the existing studies were carried out on adults subjects taking less than 5 drugs, and thus cannot be defined as taking polypharmacy according to the current consensus threshold^29^. For example, Rogers and Aronoff showed that the gut microbiota composition of adult patients chronically taking a single drug is significantly different from that of subjects without pharmacological treatments, and that the association of non-steroidal antinflammatory drugs (NSAIDs) with PPIs influences the gut microbiota in a different way than treatment with NSAIDs alone^27^. Imhann and colleagues recently demonstrated that, in adult subjects, PPIs, antibiotics, antidepressants and statins are the drug classes with the strongest influence on microbiome composition^28^. Consistently with our results, few studies carried out in adult subjects without multimorbidity have underlined that PPIs^30, 31^, antidepressants^32^, and antipsychotics^33, 34^ are the main drug classes able to induce modifications of gut microbiota composition alone.

The mechanisms of drug-induced alterations in gut microbiota are only partly known. PPIs can promote the overgrowth of specific taxa by raising luminal pH^35^, which, in turn, makes enteric mucosa more susceptible to drug-induced damage^36^. Mucosal modifications of permeability and functionality promoted by drugs can also contribute to select specific microbial taxa. For example, metformin treatment can stimulate goblet cells number and functionality, and thus contribute to select bacteria, such as *Akkermansia* spp, with a particular trophism towards mucin glycans^37^. Some drugs may also exhibit bacteriostatic or bactericidal side effects, that can selectively inhibit some bacteria and promote the overexpression of competitors. For example, metformin treatment is associated with overexpression of SCFA producers and Enterobacteriaceae through this mechanism^38^. Finally, some drugs can trigger local mucosal inflammation by raising its susceptibility to lipopolysaccharide effects, and thus contribute to select specific bacterial populations^39^.

Moreover, when a large number of drugs are co-administered in the same patient, those gut microbial taxa carrying metabolic functions that can metabolize xenobiotics may have a competitive advantage over other taxa^40, 41^. These modifications may have both positive and negative implications for the host^42^.

From the one side, they can contribute to detoxify harmful drug metabolites. On the other side, they can limit drug bioavailability and further contribute to select pathogenic taxa by modifying intestinal microenvironment^39^. Drugs that are significantly metabolized by gut bacteria include corticosteroids and statins^39^.

In our study, polypharmacy was significantly associated with the relative abundance of *Helicobacter*, which has been recently linked to the pathophysiology of a broad spectrum of extragastric diseases^43^. Similarly, the inverse association of polypharmacy with the relative abundance of members of *Lachnospiraceae* and *Succinivibrionaceae* families, producing short-chain fatty acids like butyrate, may have detrimental systemic consequences, since this microbial mediator is positively associated with cardio-respiratory fitness, colonic function, insulin-sensitivity and modulation of inflammation^12, 44, 45^.

The findings highlighted in Table 3 may be in some cases difficult to interpret, due to the poor knowledge of the microbiological and metabolic characteristics of some detected taxa. However, the current existing literature allows to make some hypothesis. For example, the positive correlation between polypharmacy and *Coprobacter* relative abundance could be explained by the presence of fermentable polysaccharides, which are among its metabolic substrates, as excipients in drug formulations^46^. Similarly, the positive association between number of drugs and *Prevotella* abundance could reflect both specific treatments and disease-related changes in lifestyle^47, 48^.

The association between polypharmacy and gut microbiota dysbiosis may have detrimental consequences for patients. This concept is supported by the increased mortality at follow-up detected in those patients who had the lowest microbiome biodiversity. Epidemiologic data have recently linked dysbiosis with increased susceptibility to systemic infections, probably as a consequence of altered mucosal permeability in the gut^24^. Drug-induced dysbiosis has been also identified as a significant predictor of hospitalization in patients with cirrhosis, by increasing the risk of hepatic encephalopathy^49, 50^. However, in our study a low Chao1 index was not associated with the risk of incident sepsis or rehospitalization. Thus, dysbiosis should be only considered a marker of poor health status, and the effects of gut microbiota composition on clinical outcomes should be better clarified in larger, population-based studies.

Surprisingly, in the present study, the gut microbiota composition of participants showed poor associations with multimorbidity and frailty. This finding is apparently inconsistent with the literature^8, 13^ and may be explained by the elevated burden of comorbidities and disability exhibited by participants. These features may in some way promote a reduced resilience or “instability” of fecal microbiota, and make it more sensitive to complex drug treatments. Thus, it can be argued that the influence of polypharmacy on gut microbiome composition is in some way mediated by multimorbidity and frailty/disability.

In this context, further studies are needed to verify the hypothesis that frailty and multimorbidity are associated with alterations in gut microbiota composition. However, the relative abundance of some taxa was significantly associated with RCFS or with CIRS Comorbidity Score. For instance, a positive association between unclassified members of *Clostridia* class and CIRS Comorbidity Score was demonstrated. This is in line with our previous research, showing that multimorbidity is an independent risk factor for the onset of *Clostridium difficile* colitis or other opportunistic infections^51, 52^.

## Conclusions

In older hospitalized patients, polypharmacy, but not multimorbidity and frailty, was significantly associated with gut microbiota dysbiosis, detected by metagenomics techniques. The severity of dysbiosis was able to significantly predict death after a 2-year follow-up. Future observational and intervention studies should better define the association between geriatric domains and gut microbiota composition, and address the complex relationship between gut microbiota and the physiopathology of aging. The effect of single drug classes on gut bacteria in elderly patients should be also better investigated in the future.

## Electronic supplementary material

Supplementary material
