# Supplementary material for: Gut microbiota composition is associated with polypharmacy in elderly hospitalized patients
Source: Sci Rep. 2017 Sep 11;7:11102. doi: 10.1038/s41598-017-10734-y (PMC5593887; doi:10.1038/s41598-017-10734-y)
Supplement: Supplementary file 1 — Supplementary material [file 41598_2017_10734_MOESM1_ESM.pdf]

SUPPLEMENTAL MATERIAL

Gut microbiota composition is associated with polypharmacy in elderly hospitalized patients

Andrea Ticinesi, Christian Milani, Fulvio Lauretani, Antonio Nouvenne, Leonardo Mancabelli, Gabriele Andrea Lugli, Francesca Turroni, Sabrina Duranti, Marta Mangifesta, Alice Viappiani, Chiara Ferrario, Marcello Maggio, Marco Ventura, Tiziana Meschi

Detail of laboratory procedures and bioinformatic analyses

Stool samples were processed and analyzed according to an established protocol of next-generation 16S rRNA microbial profiling which was developed and validated by our own research group (Milani C, et al. PlosOne 2013). The details of the laboratory procedures are reported elsewhere (Milani C, et al. Sci Rep 2016). Briefly, partial 16S rRNA gene sequences were amplified from the fecal bacterial DNA using the primer pair Probio Uni and Probio_Rev, targeting the V3 region of the 16S rRNA gene specific sequences (Milani C, et al. PlosOne 2013). Illumina adapter overhang nucleotide sequences were added to the 16S rRNA gene-specific sequences. The 16S Metagenomic Sequencing Library Preparation protocol was followed to prepare the 16S rRNA gene amplicons through the Polymerase Chain Reaction (PCR) technique. A Verity Thermocycler (Applied Biosystems Inc.) was used for this purpose. The integrity of the PCR amplicons was assessed by electrophoresis using a 2200 TapeStation Instrument (Agilent Technologies, USA). To remove primer dimers, the amplified PCR products were then purified by magnetic purification using the Agencourt AMPure XP DNA beads (Beckman Coulter Genomics GmbH, Bernried, Germany). Fluorimetric Qubit quantification system (Life Technologies) was used to esteem the DNA concentration of the amplified sequence library. Amplicons were diluted to 4 nM. The pooled final library was prepared mixing 5 µL of each diluted DNA amplicon solution. Sequencing was carried out with an Illumina MiSeq sequencer with MiSeq Reagent kit v3 chemicals.

The reads obtained by sequencing were subsequently filtered to remove low-quality and polyclonal sequences. Filtered datasets were exported as .fastq files, which were analyzed using a custom script based on the QIIME software (Caporaso JG, et al. Nat Methods 2010). Paired-end read couples (forward and reverse) were merged to reconstruct amplicons. Only sequences with a length between 140 and 400 bp and quality score >20 were considered for analyses, while sequences with homopolymers >7 bp and primers with mismatched sequence were removed. The sequences with an identity ≥97% were pooled in 16S rRNA Operational Taxonomic Units (OTUs) using the software uclust (Edgar RC. Bioinformatics 2010). OTUs with less than 10 sequences were removed from the bioinformatics analysis. All the reads were then taxonomically classified using the QIIME software suite (Caporaso JG, et al. Nat Methods 2010) and the related 16S database SILVA (Quast C, et al. Nucleic Acids Res 2013). Sample biodiversity (alpha-diversity) was calculated using the indexes of species richness Chao1 and Shannon. For each OTU assigned to a known taxon, the relative abundance in the patient microbiome was also determined. Beta diversity analysis of inter-individual variability was made with the Principal Coordinate Analysis (PCoA) method based on unweighted UniFrax method.

References

Caporaso JG, Kuczynski J, Stombaugh J, et al. QIIME allows analysis of high-throughput community sequencing data. *Nat Methods*. 2010;7(5):335-336.

Edgar RC. Search and clustering orders of magnitude faster than BLAST. *Bioinformatics*. 2010;26(19):2460-2461.

Milani C, Hevia A, Foroni E, et al. Assessing the fecal microbiota: an optimized ion torrent 16S rRNA gene-based analysis protocol. *PLoS One*. 2013;8(7):e68739.

Milani C, Ticinesi A, Gerritsen J, et al. Gut microbiota composition and Clostridium difficile infection in hospitalized elderly individuals: a metagenomic study. *Sci Rep*. 2016;6:25945.

Quast C, Pruesse E, Yilmaz P, et al. The SILVA ribosomal RNA gene database project: improved data processing and web-based tools. *Nucleic Acids Res*. 2013;41:D590-D596.

Supplemental Table. Significant correlations between relative abundance of specific taxa of gut microbiota, identified by 16S rRNA microbial profiling analyses, and clinical parameters (number of drugs, Rockwood Clinical Frailty Scale and CIRS Comorbidity Score) at multivariate linear regression models.

	Number of Drugs				Rockwood			CIRS		
	beta		SE	p*	beta	SE	p**	beta	SE	p***
Abiotrophia	-		-	-	-	-	-	-	-	-
Acetaerobacterium	-		-	-	-	-	-	-	-	-
Acidaminococcus	-		-	-	-	-	-	-	-	-
Actinobacillus	-		-	-	-	-	-	-	-	-
Actinomyces	-		-	-	-	-	-	-	-	-
Akkermansia	-		-	-	-	-	-	-	-	-
Albidiferax	-		-	-	-	-	-	-	-	-
Alistipes	-0.006	±	0.003	0.03	-	-	-	-	-	-
Allisonella	-		-	-	-	-	-	-	-	-
Alloprevotella	-		-	-	-	-	-	-	-	-
Alloscardovia	-		-	-	-	-	-	-	-	-
Aquabacterium	-		-	-	-	-	-	-	-	-
Aquamicrobium	-		-	-	-	-	-	-	-	-
Asteroleplasma	0.0001	±	0.00004	0.05	-	-	-	-	-	-
Atopobium	-		-	-	-	-	-	-	-	-
Bacteroides	-		-	-	-	-	-	-	-	-
Barnesiella	-0.001	±	0.0006	0.05	-	-	-	-	-	-
Bifidobacterium	-		-	-	-	-	-	-	-	-
Bilophila	-0.00008	±	0.00004	0.04	-	-	-	-	-	-
Blautia	-		-	-	-	-	-	-	-	-
Brachyspira	-		-	-	-	-	-	-	-	-
Bradyrhizobium	0.000002	±	0.0000001	0.01	-	-	-	-	-	-
Brevibacterium	-		-	-	-	-	-	-	-	-

Brevundimonas	-		-	-	-	-	-	-	-	-
Butyricicoccus	-		-	-	-	-	-	-	-	-
Butyricimonas	-		-	-	-	-	-	-	-	-
Butyrivibrio	-		-	-	-	-	-	-	-	-
Campylobacter	0.0004	+	0.0002	0.04	-	-	-	-	-	-
Catenibacterium	-		-	-	-	-	-	-	-	-
Cellulosilyticum	-		-	-	-	-	-	-	-	-
Christensenella	-		-	-	-	-	-	-	-	-
Citrobacter	-		-	-	-	-	-	-	-	-
Cloacibacillus	-		-	-	-	-	-	-	-	-
Clostridium sensu stricto 3	-		-	-	-	-	-	-	-	-
Clostridiales bacterium 20-2a	-		-	-	-	-	-	-	-	-
Clostridium sensu stricto 1	-		-	-	-	-	-	-	-	-
Collinsella	-		-	-	-	-	-	-	-	-
Coprobacillus	-		-	-	-	-	-	-	-	-
Coprobacter	0.0004	+	0.0002	0.03	-	-	-	-	-	-
Coprococcus	-		-	-	-	-	-	-	-	-
Corynebacterium	-0.0008	+	0.00004	0.04	-	-	-	-	-	-
Candidatus _Controlccharimonas	-		-	-	-	-	-	-	-	-
Candidatus Arthromitus	-		-	-	-	-	-	-	-	-
Candididate division TM7 genomosp. P1	-		-	-	-	-	-	-	-	-
Desulfovibrio	-		-	-	-	-	-	-	-	-
dgA-11 gut group	-		-	-	-	-	-	-	-	-
Dialister	-		-	-	-	-	-	-	-	-
Dorea	-		-	-	-	-	-	-	-	-
Eggerthella	-0.00003	+	0.000001	0.05	-	-	-	-	-	-
Enhydrobacter	-		-	-	-	-	-	-	-	-
Enterobacter	0.00002	+	0.00001	0.04	-	-	-	-	-	-
Enterococcus	-		-	-	-	-	-	-	-	-
Enterorhabdus	-		-	-	-	-	-	-	-	-
Epulopiscium	-		-	-	-	-	-	-	-	-
Escherichia-Shigella	-		-	-	-	-	-	-	-	-
Faecalibacterium	-		-	-	-	-	-	-	-	-

Finegoldia	-	-	-	-	-	-	-	-	-
Flavobacterium	-	-	-	-	-	-	-	-	-
Flavonifractor	-	-	-	-	-	-	-	-	-
Fonticella	0.0009 ±	0.00004	0.04	-	-	-	-	-	-
Fusobacterium	-	-	-	-	-	-	-	-	-
Gelria	-	-	-	-	-	-	-	-	-
Gemella	-	-	-	-	-	-	-	-	-
Gordonibacter	-	-	-	-	-	-	-	-	-
Haemophilus	-	-	-	-	-	-	0.00004 ±	0.00002	0.03
Hafnia	-	-	-	-	-	-	0.00006 ±	0.000003	0.01
Halaerobium	-	-	-	-	-	-	-	-	-
Helicobacter	0.0006 ±	0.00002	0.009	-	-	-	-	-	-
Holdemania	-	-	-	-	-	-	-	-	-
Howardella	-	-	-	-	-	-	-	-	-
Hydrogenophaga	-0.00008 ±	0.00004	0.05	-	-	-	-	-	-
Hydrogenoaerobacterium	-	-	-	-	-	-	-	-	-
Hyphomicrobium	-	-	-	-	-	-	-	-	-
Intestinimonas	-	-	-	-	-	-	-	-	-
Athinobacterium	-	-	-	-	-	-	-	-	-
Klebsiella	-	-	-	-	-	-	-	-	-
Lachnospira	-	-	-	-	-	-	-	-	-
Lactobacillus	-	-	-	-	-	-	-	-	-
Lactococcus	-	-	-	-	-	-	-	-	-
Leptothrix	-	-	-	-	-	-	-	-	-
Marinilactibacillus	-	-	-	-	-	-	-	-	-
Marvinbryantia	-	-	-	-	-	-	-	-	-
Massilia	-	-	-	-	-	-	-	-	-
Megamonas	-	-	-	-	-	-	-	-	-
Megasphaera	-	-	-	-	-	-	-	-	-
Methobrevibacter	-	-	-	-	-	-	-	-	-
Mitsuokella	-	-	-	-	-	-	-	-	-
Mogibacterium	-	-	-	-	-	-	-	-	-
Morganella	0.00004 ±	0.00001	0.003	-	-	-	-	-	-

Moryella	-		-	-	-		-	-	-		-	-
Mucispirillum	-		-	-	-		-	-	-		-	-
Nitrospira	-		-	-	-		-	-	-		-	-
Odoribacter	-		-	-	-		-	-	-		-	-
Olsenella	-		-	-	-		-	-	-		-	-
Oscillibacter	-		-	-	-		-	-	-		-	-
Oscillospira	-		-	-	0.0005	±	0.00002	0.03	-		-	-
Papillibacter	-		-	-	-		-	-	-		-	-
Parabacteroides	-		-	-	-		-	-	-		-	-
Paraprevotella	-		-	-	-		-	-	-		-	-
Parasutterella	-		-	-	-		-	-	-		-	-
Parvimonas	-		-	-	-		-	-	-		-	-
Parvularcula	-		-	-	-		-	-	-		-	-
Peptococcus	-		-	-	-		-	-	-		-	-
Petrimonas	-		-	-	-		-	-	-		-	-
Phascolarctobacterium	-		-	-	-		-	-	-		-	-
Porphyromonas	-		-	-	-		-	-	-		-	-
Prevotella	0.003	±	0.0001	0.02	0.00001	±	0.000006	0.02	-		-	-
Propionibacterium	-		-	-	-		-	-	0.00001	±	0.000005	0.01
Proteiniphilum	-		-	-	-		-	-	-		-	-
Poteus	-		-	-	-		-	-	-		-	-
Pseudoalteromonas	-		-	-	-		-	-	-		-	-
Pseudobutyrivibrio	-		-	-	-		-	-	-		-	-
Pseudoflavonifractor	-		-	-	-		-	-	-		-	-
Pseudomonas	-		-	-	-		-	-	-		-	-
Psychrobacter	-		-	-	-		-	-	-		-	-
Pyramidobacter	-		-	-	-		-	-	-		-	-
Pantoea	-		-	-	-		-	-	0.0002	±	0.00008	0.01
RC9 gut group	-		-	-	-		-	-	-		-	-
Rikenella	-		-	-	-		-	-	-		-	-
Robinsoniella	-		-	-	-		-	-	-		-	-
Roseburia	-		-	-	-		-	-	-		-	-
Rothia	-		-	-	-		-	-	-		-	-

Ruminococcus	-	-	-	-	-	-	-	-	-
Scardovia	-	-	-	-	-	-	0.000003 ±	0.00000008	0.05
Schwartzia	-	-	-	-	-	-	-	-	-
Sedimentibacter	-	-	-	-	-	-	-	-	-
Shuttleworthia	-	-	-	-	-	-	-	-	-
Slackia	-	-	-	-	-	-	-	-	-
SP3-e08	-	-	-	-	-	-	-	-	-
Staphylococcus	-	-	-	-	-	-	-	-	-
Streptococcus	-	-	-	-	-	-	-	-	-
Subdoligranulum	-	-	-	-	-	-	-	-	-
Succiniclasticum	-	-	-	-	-	-	0.00001 ±	0.000001	0.05
Succinivibrio	-	-	-	-	-	-	-	-	-
Sutterella	-	-	-	-	-	-	-	-	-
Synergistes	-	-	-	-	-	-	-	-	-
Thalassospira	-0.0005 ±	0.0002	0.04	-	-	-	-	-	-
TM7 *phylum* sp. oral clone EW055	-	-	-	-	-	-	-	-	-
Turicibacter	-	-	-	-	-	-	-	-	-
Undibacterium	-	-	-	-	-	-	-	-	-
U. m. of Rikenellaceae family	0.00003 ±	0.000001	0.05	-	-	-	-	-	-
U. m. of S24-7 family	-	-	-	-	-	-	-	-	-
U. m. of Bacteroidales order	-	-	-	-	-	-	-	-	-
U. m. of Cytophagaceae family	-	-	-	-	-	-	-	-	-
U. m. of Chitinophagaceae family	-	-	-	-	-	-	-	-	-
U. m. of Bacteroidetes phylum	-	-	-	-	-	-	-	-	-
U. m. of Candidate division TM7 phylum	-	-	-	-	-	-	-	-	-
U. m. of KD4-96 class	-	-	-	-	-	-	-	-	-
U. m. of Gastranaerophilales order	-	-	-	-	-	-	-	-	-
U. m. of Bacillaceae family	-	-	-	-	-	-	-	-	-
U. m. of Ll142-3M24 family	-	-	-	-	-	-	-	-	-
U. m. of P5D1-392 family	-	-	-	-	-	-	-	-	-
U. m. of Lactobacillales order	-	-	-	-	-	-	-	-	-
U. m. of Bacilli class	-	-	-	-	-	-	-	-	-
U. m. of Christensenellaceae family	-	-	-	-	-	-	-	-	-

U. m. of Clostridiaceae 1 family	-		-	-	0.00002	+	0.00001	0.05	-		-	-
U. m. of Defluviitaleaceae family	-		-	-	-		-	-	-		-	-
U. m. of Eubacteriaceae family	-		-	-	-		-	-	-		-	-
U. m. of Family XIII family (Clostridial	-		-	-	-		-	-	-		-	-
U. m. of Family XI family (Clostridiales	-		-	-	-		-	-	-		-	-
U. m. of Lachnospiraceae family	-0.13	+	0.006	0.03	-		-	-	-		-	-
U. m. of Peptococcaceae family	-		-	-	-		-	-	-		-	-
U. m. of Peptostreptococcaceae family	-		-	-	-		-	-	-		-	-
U. m. of Ruminococcaceae family	0.005	+	0.002	0.02	-		-	-	-		-	-
U. m. of vadinBB60 family	-		-	-	-		-	-	-		-	-
U. m. of Clostridiales order	-		-	-	-		-	-	-		-	-
U. m. of Clostridia class	-		-	-	-		-	-	-		-	-
U. m. of Erysipelotrichaceae family	-		-	-	-		-	-	-0.0007	+	0.00004	0.05
U. m. of Erysipelotrichaceae family (Ery	-		-	-	-		-	-	0.00005	+	0.00001	<.0001
U. m. of Veillonellaceae family	-		-	-	-		-	-	-		-	-
U. m. of Firmicutes phylum	-		-	-	-		-	-	-		-	-
U. m. of CFT112H7 family	-		-	-	-		-	-	-		-	-
U. m. of Fusobacteriales order	-		-	-	-		-	-	-		-	-
U. m. of Caulobacteraceae family	-		-	-	-		-	-	-		-	-
U. m. of A0839 family	0.00008	+	0.00003	0.02	-		-	-	-		-	-
U. m. of Rhodospirillales order	-		-	-	-		-	-	-		-	-
U. m. of Rickettsiales order	-		-	-	-		-	-	-		-	-
U. m. of LD12 freshwater group family	-		-	-	-		-	-	-		-	-
U. m. of Sphingomonadaceae family	-		-	-	-		-	-	-		-	-
U. m. of Sphingomonadales order	-		-	-	-		-	-	-		-	-
U. m. of Alphaproteobacteria class	-		-	-	-		-	-	-		-	-
U. m. of Comamonadaceae family	-		-	-	-		-	-	-		-	-
U. m. of Oxalobacteraceae family	-		-	-	-		-	-	-		-	-
U. m. of Burkholderiales order	-		-	-	-		-	-	-		-	-
U. m. of Betaproteobacteria class	-		-	-	-		-	-	-		-	-
U. m. of Desulfovibrionaceae family	0.000001	+	0.0000003	0.02	-		-	-	-		-	-
U. m. of Succinivibrionaceae family	-		-	-	-		-	-	-		-	-
U. m. of Enterobacteriaceae family	-		-	-	-		-	-	-		-	-

U. m. of Gammaproteobacteria class	-	-	-	-	-	-	-	-	-
U. m. of Proteobacteria phylum	-	-	-	-	-	-	-	-	-
U. m. of NB1-n order	-	-	-	-	-	-	-	-	-
U. m. of RF9 order	-	-	-	-	-	-	-	-	-
U. m. of vadinHA64 order	-	-	-	-	-	-	-	-	-
U. m. of Bacteria kingdom	-	-	-	-	-	-	-	-	-
U. m. of Acidimicrobiales order	-	-	-	-	-	-	-	-	-
U. m. of Bifidobacteriaceae family	-	-	-	-	-	-	-	-	-
U. m. of Corynebacteriaceae family	-	-	-	-	-	-	-	-	-
U. m. of Actinobacteria class	-	-	-	-	-	-	-	-	-
U. m. of Coriobacteriaceae family	-	-	-	-	-	-	-	-	-
U. m. of Actinobacteria phylum	-	-	-	-	-	-	-	-	-
U. m. of Porphyromonadaceae family	-	-	-	-	-	-	-	-	-
U. of Prevotellaceae family	-	-	-	-	-	-	-	-	-
U. m. of rat_AT060301C family	-	-	-	-	-	-	-	-	-
U. m. of Halobacteriaceae family	-	-	-	-	-	-	-	-	-
Vagococcus	-	-	-	-	-	-	-	-	-
Variovorax	-	-	-	-	-	-	-	-	-
Veillonella	-	-	-	-	-	-	-	-	-
Victivallis	-	-	-	-	-	-	-	-	-

U. m. = Unclassified member; SE = Standard Error; CIRS = Cumulative Illness Rating Scale.

p*= Multivariate linear regression models adjusted for age, sex, Rockwood Clinical Frailty Scale, CIRS Comorbidity Score.

p**= Multivariate linear regression models adjusted for age, sex, number of drugs, CIRS Comorbidity Score.

p***= Multivariate linear regression models adjusted for age, sex, number of drugs, Rockwood Clinical Frailty Scale.

Supplemental figure 1.

Taxonomic analysis of gut microbiota. Overall composition of gut microbiota at genus level, sample by sample, detected by 16S rRNA microbial profiling.

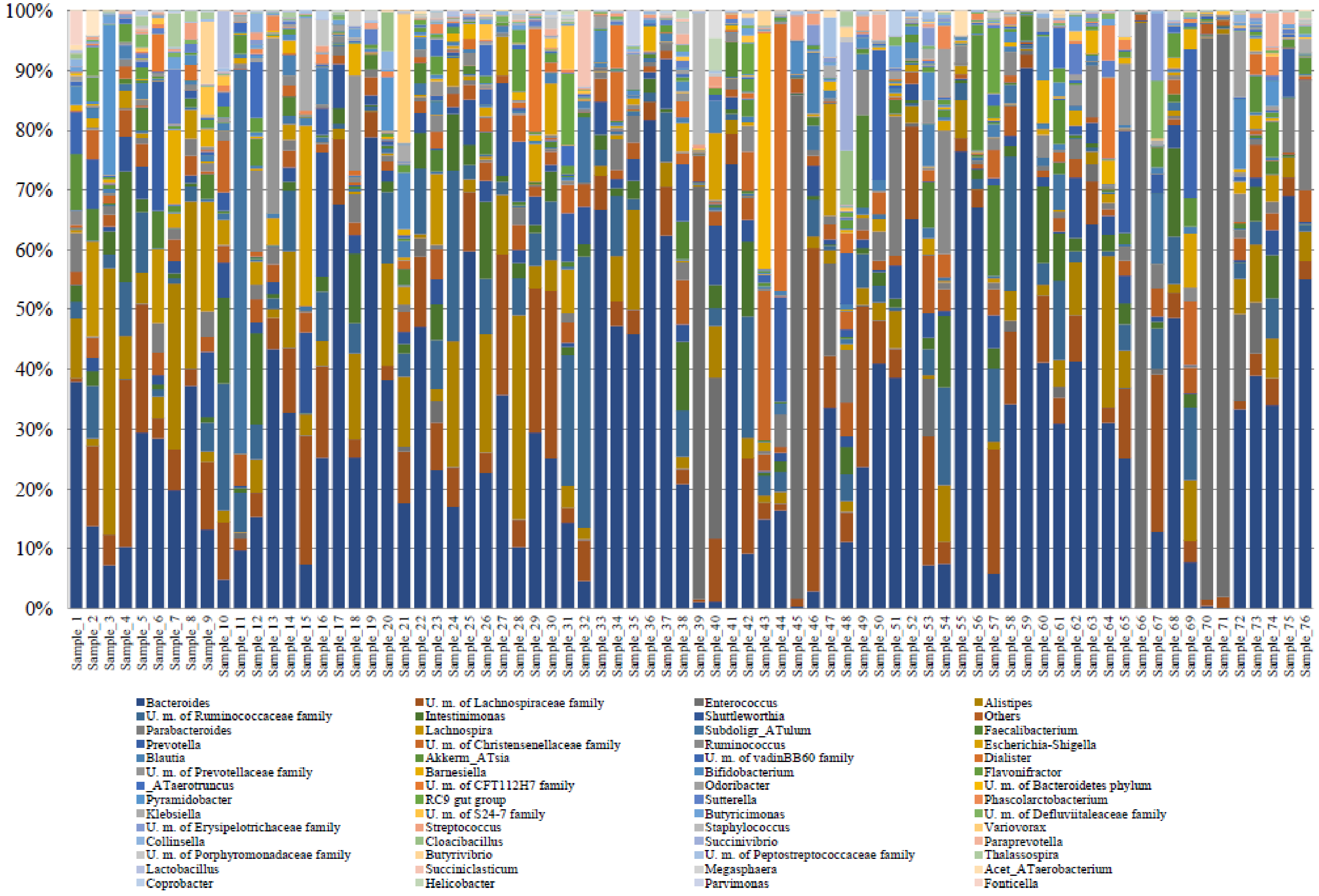

Supplemental figure 2.

Effect of single drug class exposure on the overall composition of fecal microbiota. Principal Coordinate Analyses based on unweightd UniFrax showing inter-individual variability of fecal microbiota composition according to the chronic exposure to single drug classes.

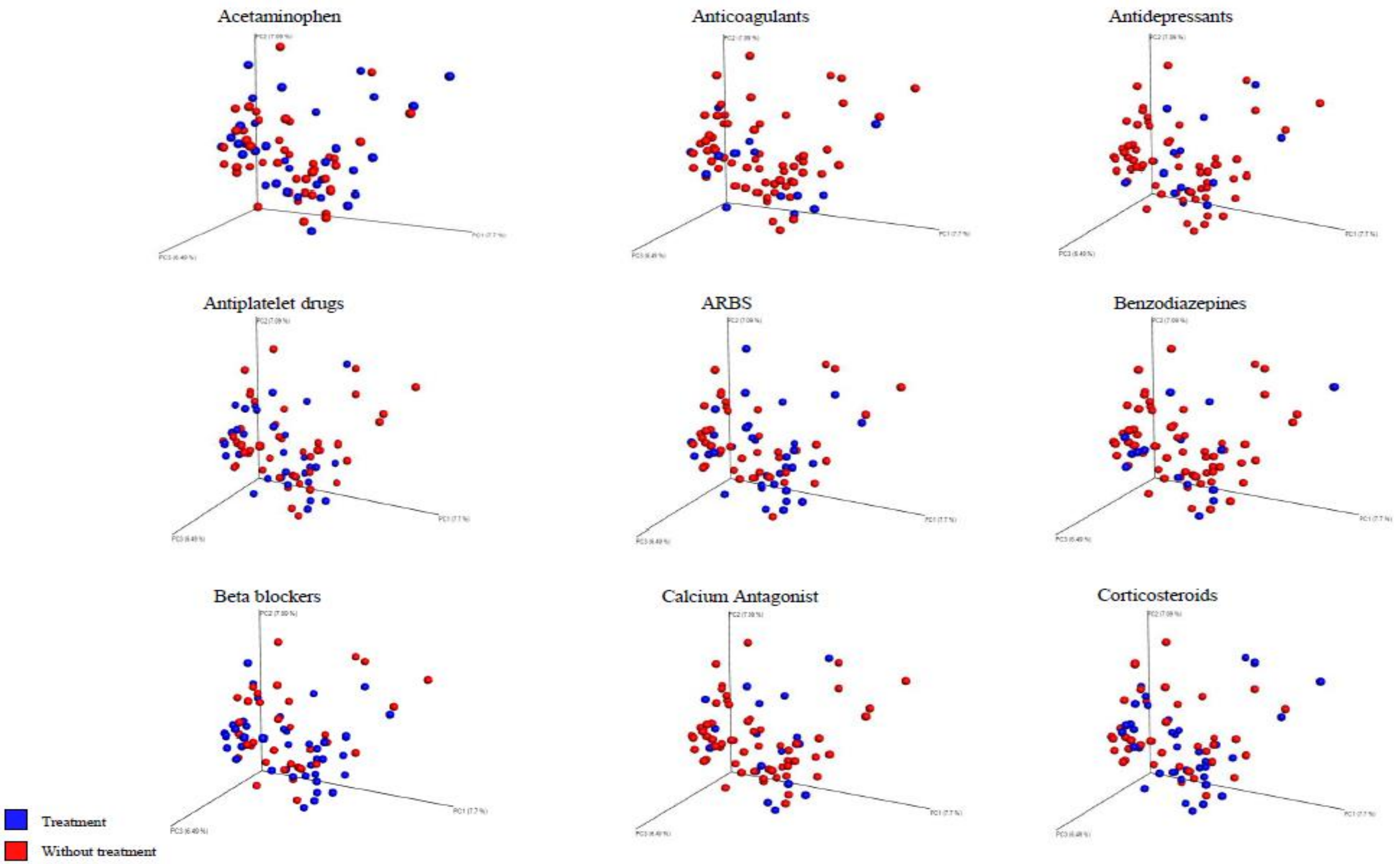

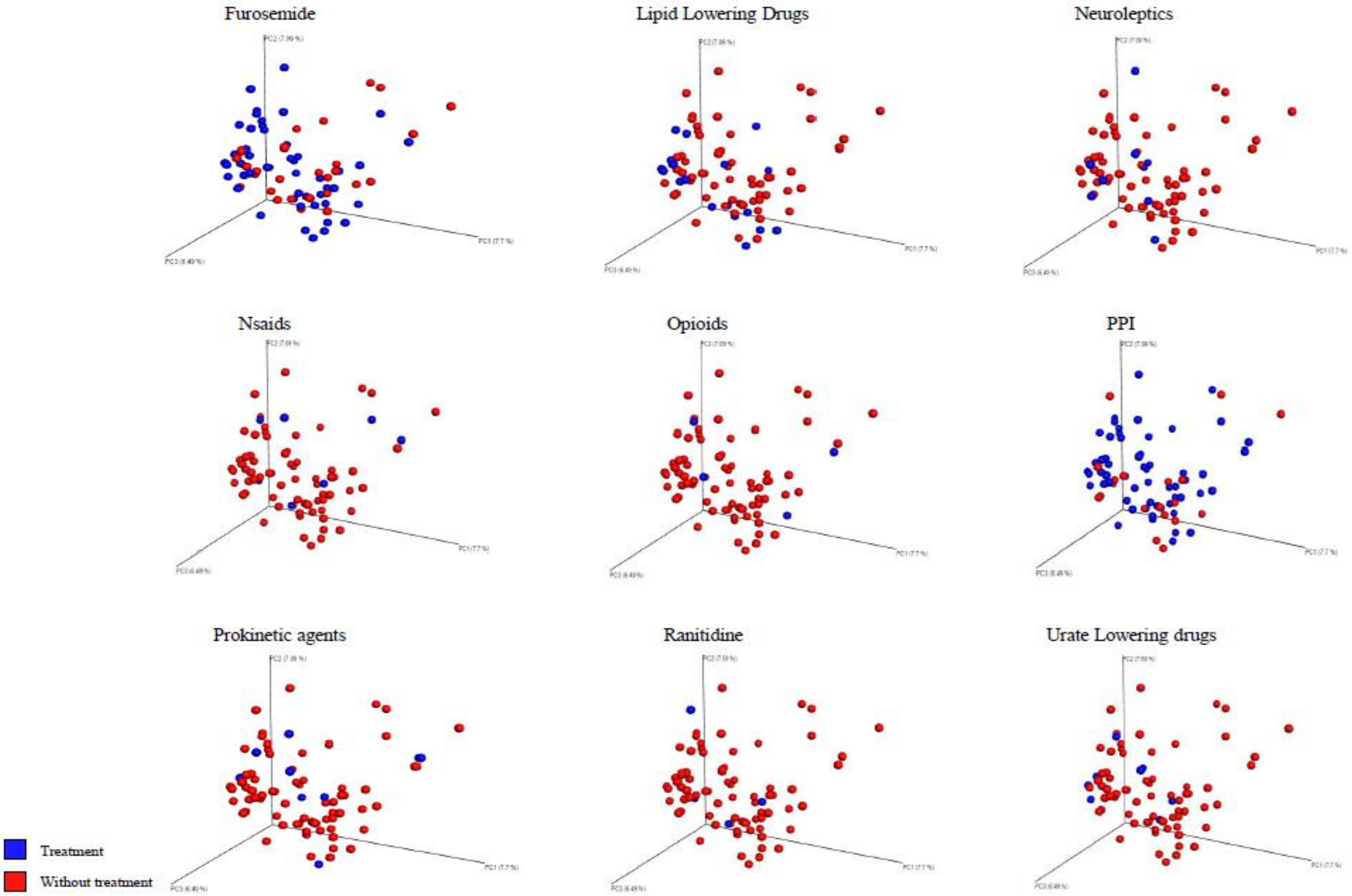
Furosemide
Lipid Lowering Drugs
Neuroleptics
Nsaids
Opioids
PPI
Prokinetic agents
Ranitidine
Urate Lowering drugs
Treatment
Without treatment
